# Assessment of Knowledge, Attitudes, and Willingness Among People With and Without Heart Disease Relatives in Saudi Arabia Regarding Cardiopulmonary Resuscitation: A Cross-Sectional Study

**DOI:** 10.7759/cureus.51197

**Published:** 2023-12-27

**Authors:** Hamdan AlShehri, Saleh M AlKulayb, Hatim S Karhan, Ebtehag A Al wargash, Rana M Althobaiti, Masoud H Alsinan, Hajar A Al mustanyir, Ghadah Al-swat, Razan A Almakki, Renad M Alammari

**Affiliations:** 1 Internal Medicine, Najran University, Najran, SAU; 2 Medicine, Najran University, Najran, SAU; 3 Medicine, Taif University, Taif, SAU; 4 College of Medicine, Najran University, Najran, SAU

**Keywords:** kingdom of saudi arabia (ksa), assessment of heart disease, cpr knowledge, heart disease and cpr, cpr

## Abstract

Background

Cardiopulmonary resuscitation (CPR) is a life-saving procedure that can be performed in many situations where a person's breathing or heartbeat has stopped, such as during a heart attack, suffocation, near drowning, or electrical injuries.

Despite its importance, to our knowledge, no research has been conducted yet in our community on the differences in CPR knowledge, attitudes, and willingness between those with and without heart disease relatives.

Objective

This study aimed to assess the level of knowledge, attitude, and willingness of people with and without heart disease relatives to perform CPR in a case of cardiac arrest.

Methods

A descriptive cross-sectional study was carried out between July 2023 and October 2023 among the Saudi Arabia population using a self-administered online questionnaire focusing on the sociodemographic characteristics of participants and the level of knowledge, attitude toward CPR, and the willingness to perform CPR in a case of cardiac arrest.

Results

This study included 799 participants, with 331 males (41.4%) and 468 females (58.6%). Age distribution was mainly in the range of 18-25 years (241 participants, 30.2%). Geographically, the highest proportion was from the southern region (214 participants, 26.8%). The majority had a university degree (533 participants, 66.7%). Employment status varied, with 401 participants (50.2%) working outside the medical field. Monthly income showed that 297 (37.2%) had an income ranging from 2,000 to 10,000 SAR.

The average daily sleep duration varied, with the majority (64.7%) reporting six to nine hours of sleep. Also, 44.2% of participants reported exercising. Regarding smoking status, 80.9% reported not smoking. A family history of heart disease was reported by 46.4% of participants, whereas 16.3% reported a personal diagnosis of heart disease.

Regarding knowledge, 40.8% correctly identified when CPR should be performed. Only 40.4% correctly identified the first step in a CPR situation, and only 22.9% identified the correct sequence of steps for performing CPR. Additionally, only 66.5% correctly identified the emergency hotline number "Red Crescent." Furthermore, only 8.9% knew the correct compression/ventilation ratio for adults during CPR.

As for the attitude, a significant percentage (65.0%) had not taken a CPR course. However, the majority (84.6%) expressed a strong desire to learn CPR. Interestingly, the participants showed a positive attitude toward CPR education.

Among the participants, 53.9% responded that they performed CPR when encountering a situation that required it, while 46.1% did not. Regarding the participants' willingness to perform CPR on different individuals, the majority (74.1%) expressed their readiness to perform CPR whenever needed.

Conclusion

The findings demonstrated knowledge gaps, with misconceptions about CPR. However, participants generally expressed a positive attitude toward CPR education and a willingness to learn. Factors such as age showed a weak association with knowledge level, while gender, region, education, employment, and family history of heart disease did not significantly impact knowledge. The study highlighted the need for improved CPR education and awareness. Hence, we recommend CPR courses to be implemented as a graduation prerequisite. In addition to that, linking some government services to a first aid course would positively impact the general population's practices in CPR and other emergencies.

## Introduction

Cardiopulmonary resuscitation (CPR) is a life-saving procedure that can be carried out in many situations where a person's breathing or heartbeat has stopped, such as during a heart attack, suffocation, near drowning, or electrical injuries [1]. The American Heart Association suggests establishing CPR with firm, rapid chest compressions. It is advised that both untrained bystanders and medical professionals perform hands-only CPR [2]. Kouwenhoven et al. said, "Anyone, anytime, can now initiate cardiac resuscitative measures. All that is needed are two hands" [3]. These were the first words that referred to chest cardiac massage in 1960; at the time, it was thought to be a novel method for providing cardiac massage without a thoracotomy.

Globally, sudden cardiac arrest (SCA) is a serious issue for public health, mostly affecting patients with heart disease. Heart diseases remain the chief cause of cardiac arrest in comparison with non-cardiac causes [4]. In Saudi Arabia, 74% of SCAs occurred at home, with a minority of bystander CPR performed and family members present before emergency medical services (EMS) arrived [5]. Thus, bystander CPR is the single most important factor in the survival of out-of-hospital cardiac arrest victims.

Regarding knowledge that can save lives and the skills necessary to use it, public awareness is crucial. Numerous studies have been conducted to assess the CPR knowledge and training status of people who are not involved in health care in various communities. Only 28.7% of respondents in a Jeddah-based Saudi study reported having had CPR instruction in the past, compared to 25.6% in China, 29% in Jordan, 40.7% in Izmir, and 55.7% in Australia. About 83.2% in Germany and 64% in Canada and many nations, like Slovenia and Japan, have made basic life support (BLS) training courses a requirement for obtaining a driver's license [6].

In a study conducted among university students in Riyadh, Saudi Arabia, 31% of students had no prior experience with CPR according to research measuring knowledge and attitudes about CPR. About 85% of those who had prior information thought it was insufficient. Approximately 13% of people had encountered a situation requiring CPR, but only 14% of them performed it. This is mainly brought on by a lack of education (48.2%). In all, 88% of students expressed a desire to learn CPR. The authors concluded that while there was a generally good attitude toward CPR, there was an inadequate understanding of the subject [7].

In another study from Doha, Qatar, 2.4% of all hospital admissions had greater survival rates among patients who had out-of-hospital cardiac arrest (OHCA) and bystander CPR [8]. Bystanders in developed nations find it challenging to do CPR owing to a lack of training, abilities, confidence issues, and fear of legal repercussions for delivering subpar care [9].

The knowledge and training among Saudi Arabian individuals are inadequate. There was a lack of public awareness and knowledge of CPR, although the public was willing to increase their CPR knowledge and abilities [6]. There is a significant impact of having good public awareness about how to practice CPR.

In most OHCA situations, the first person to intervene is a victim's relative, who usually is a non-medical person [10].

Despite its importance, to our knowledge, no study has been conducted on the difference in CPR knowledge, attitudes, and willingness between those with and without heart disease relatives in Saudi Arabia. Therefore, this study was conducted to assess knowledge, attitudes, and willingness toward CPR among people with and without heart disease relatives in cases of cardiac arrest among the Saudi population across different regions of Saudi Arabia.

## Materials and methods

This study is a descriptive cross-sectional study and was carried out between July 2023 and October 2023 among the general population of Saudi Arabia. Participants less than 18 years of age or those who are students or work in the medical field were also excluded from the study. The sample size was calculated using the online sample size calculator provided by RaoSoft. A minimum of 267 participants were required for this study to be completed, taking into consideration a maximum uncertainty of 50% of positive responses, a maximum error of 5%, and a 90% confidence level. The study was approved by the Research and Ethical Committee of Najran University (Reference No.: 012956-029278-DS).

The study questionnaire was adopted from a study conducted in Saudi Arabia at King Saud University with some modifications mainly by adding more of the sociodemographic variables to be studied, and validity was established using outside source experts in CPR training. Also, a pilot study of 20 participants was conducted before the data collection process for insights. The questionnaire was a self-administered online questionnaire that was distributed among participants and included 26 questions all of which were in Arabic and English languages via Google Forms (Google, Mountain View, CA). The information in the questions covered mainly two domains, which were questions related to the sociodemographic characteristics of participants and questions related to the level of knowledge, attitude towards CPR, and willingness to perform CPR in cases of cardiac arrest.

Each response was linked to one Google account to avoid repeated responses without breaching the anonymity of the participants. In addition, to minimize errors, a contact number for any inquiries or needs for more explanation regarding any questions was provided in the questionnaire. Our questionnaire was distributed through varieties of channels to reach the target populations, which included but was not limited to distributing links in social media and QR codes at malls, universities, and mosques.

Once the data was extracted, it was reviewed, coded, and introduced into IBM SPSS version 28 for statistical analysis. Descriptive analysis was performed using frequency and percent distributions for all variables, including participants' demographic information, which included gender, age, education, occupation, monthly income, residence, sleeping hours, exercise habits, and smoking habits. Also, past specific cardiac antecedents, family, and social histories among the study participants and their relatives will be investigated.

To assess the knowledge regarding CPR, the knowledge was scored using the Saudi Heart Association's guidelines and recommendations regarding CPR and was calculated by adding up the scores for each item. Correct responses were given one point and incorrect answers or inadequate information were given zero points. Participants who scored less than 50% of the maximum score were classified as having a poor knowledge level, while those who scored 50% or more were classified as having a good knowledge level.

To assess factors associated with participants' knowledge level about CPR, a cross-tabulation graph was used, and Pearson's chi-square test and Fisherman's exact test for small frequency distributions were performed, and a value of less than 0.05 was considered to be statistically significant.

Additionally, participants' knowledge regarding CPR was tabulated, and their overall knowledge level and source of information were graphed.

## Results

**Figure 1:**
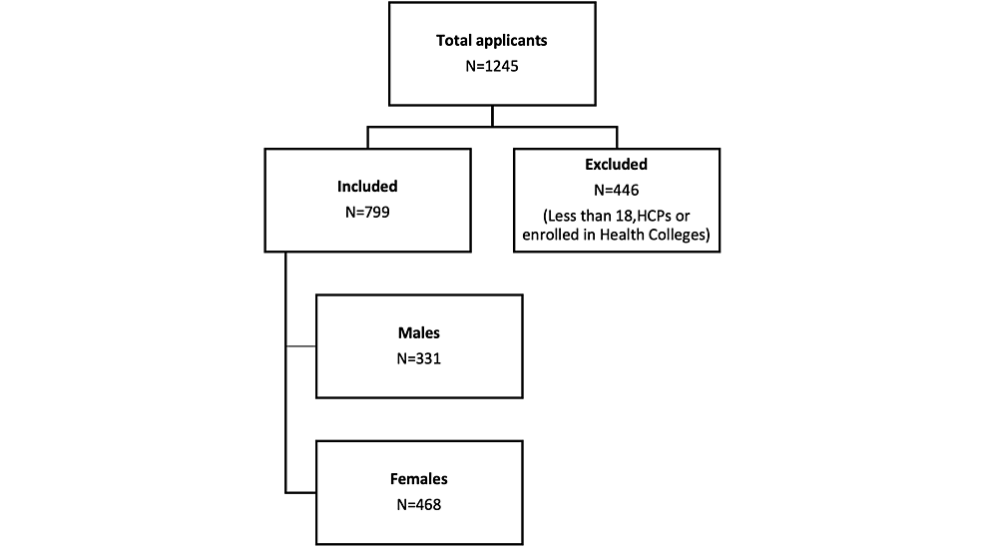
Enrollment of study participants HCPs: Healthcare professionals.

A total of 1245 individuals applied to participate. Out of these applicants, 799 individuals were included in the study, while 446 individuals were eliminated. The reasons for elimination were primarily due to the individuals being less than 18 years old, healthcare professionals (HCPs), or already enrolled in health colleges. These criteria were established to ensure that our study focused on individuals without professional training or education in healthcare, allowing us to assess the general population's knowledge, attitude, and willingness to perform CPR (Figure 1).

The results from Table 1 indicate that the sample consisted of 331 males (41.4%) and 468 females (58.6%). Age distribution showed a significant presence of participants in the range of 18-25 years (241 participants, 30.2%), followed by those in the range of 41-50 years (159 participants, 19.9%). Geographically, the highest proportion of participants was from the southern region (214 participants, 26.8%), while the Eastern Province had the lowest representation (92 participants, 11.5%). In terms of education, the majority of participants had a university degree (533 participants, 66.7%), while 245 participants (30.7%) had a high school education. Employment status varied, with 401 participants (50.2%) working outside the medical field, 142 participants (17.8%) being non-medical field students, and 256 participants (32.0%) not working. The monthly income distribution showed that 297 participants (37.2%) had an income ranging from 2000 to 10,000 SAR, while 266 participants (33.3%) had a monthly income of less than 2000 SAR.

**Table 1:** Sociodemographic data of study participants The data have been represented as numbers (N) and percentages (%).

Parameters	Category	Count (N = 799)	%
Gender	Male	331	41.4%
	Female	468	58.6%
Age grouping	18-25 years	241	30.2%
	26-30 years	104	13.0%
	31-35 years	86	10.8%
	36-40 years	118	14.8%
	41-50 years	159	19.9%
	51-60 years	71	8.9%
	More than 60 years	20	2.5%
Region	Central Region	137	17.1%
	Western Region	169	21.2%
	Northern Region	187	23.4%
	Southern Region	214	26.8%
	Eastern Province	92	11.5%
Educational status	Primary	8	1.0%
	Intermediate	13	1.6%
	High school	245	30.7%
	University	533	66.7%
Employment status	I work but not in the medical field	401	50.2%
	I am a student but not in the medical field	142	17.8%
	I don’t work	256	32.0%
Monthly income	Less than 2000 SAR	266	33.3%
	2000-10,000 SAR	297	37.2%
	10,000-20,000 SAR	203	25.4%
	More than 20,000 SAR	33	4.1%

The social, family, and medical histories of the study participants are summarized in Table 2. The average daily sleep duration among participants varied, with the majority (64.7%) reporting six to nine hours of sleep. A smaller percentage reported sleeping for three hours or less (0.9%), three to five hours (26.2%), or more than nine hours (8.3%). In terms of exercise habits, 44.2% of participants reported exercising, while 55.8% did not. Among those who exercised, the frequency varied, with 22.9% exercising once a week, 40.8% exercising two to three times a week, 17.3% exercising four to five times a week, and 19.0% almost exercising daily. Regarding smoking status, the majority of participants (80.9%) reported not smoking, while 13.1% were current smokers and 6.0% were former smokers. Family history of heart disease was reported by 46.4% of participants, with the most common kinship being father (26.7%) and mother (21.8%). Among the participants, 16.3% reported a personal diagnosis of heart disease, with the most common specific diagnoses being myocardial infarction (23.1%) and hypertension (40.8%), followed by arrhythmia (15.4%), congenital heart disease (5.4%), dyslipidemia (6.9%), and cardiomyopathies (8.5%).

**Table 2:** Social, family, and medical histories of the study's participants The data have been represented in numbers (N) and percentages (%)

Parameters	Category	Count (N = 799)	%
Average daily sleep duration	3 hours or less	7	0.9%
	3-5 hours	209	26.2%
	6-9 hours	517	64.7%
	More than 9 hours	66	8.3%
Exercise habits	No	446	55.8%
	Yes	353	44.2%
Frequency of weekly exercise	Once	81	22.9%
	2-3 times	144	40.8%
	4-5 times	61	17.3%
	I almost exercise daily	67	19.0%
Smoking status	Current smoker	105	13.1%
	Former smoker	48	6.0%
	I do not smoke	646	80.9%
Family history of heart disease	No	428	53.6%
	Yes	371	46.4%
Kinship of family member with heart disease (if applicable)	Father	99	26.7%
	Mother	81	21.8%
	Brother	32	8.6%
	Sister	29	7.8%
	Uncle	53	14.3%
	Aunt	25	6.7%
	Grandfather	27	7.3%
	Grandmother	25	6.7%
Personal diagnosis of heart disease	No	669	83.7%
	Yes	130	16.3%
Specific heart disease diagnosis (if applicable)	Myocardial infarction (heart attack)	30	23.1%
	Arrhythmia	20	15.4%
	Congenital heart disease (birth defects)	7	5.4%
	Dyslipidemia (High blood fat)	9	6.9%
	Hypertension	53	40.8%
	Cardiomyopathies	11	8.5%

The assessment of knowledge regarding CPR among the study participants revealed some gaps in understanding. Among the participants, 40.8% correctly identified that CPR should be performed when a person's breathing or heartbeat has stopped. However, a notable percentage believed that CPR should only be done when a person's breathing has stopped (9.4%) or when a person's heartbeat has stopped (15.5%). In terms of the first step in a CPR situation, 40.4% correctly stated that calling an ambulance is the first thing to do. However, a significant percentage believed that starting chest compressions was the initial step (28.3%). When asked about the correct sequence of steps for performing CPR, only 22.9% identified the correct order of maintaining a patent airway, performing compressions, and then performing artificial breathing. Additionally, there was some confusion regarding the emergency hotline number "Red Crescent," with 66.5% correctly identifying it as 997. Furthermore, only 8.9% knew the correct compression/ventilation ratio for adults during CPR as 30:2 (Table 3).

**Table 3:** Assessment of knowledge regarding cardiopulmonary resuscitation among study participants The data have been represented in numbers (N) and percentages (%). CPR: Cardiopulmonary resuscitation.

Parameters	Category	Count (N = 799)	%
In which of the following situations should CPR be done?	A person's breathing only has stopped	75	9.4%
	A person's heartbeat only has stopped	124	15.5%
	A person's breathing or heartbeat has stopped	326	40.8%
	I don’t know	274	34.3%
What is the FIRST thing to do if you encounter a situation requiring CPR?	Call an ambulance	323	40.4%
	Start chest compressions	226	28.3%
	Take the victim to the nearest hospital	97	12.1%
	I don’t know	153	19.1%
The correct sequence of the steps for performing CPR is:	Maintain a patent airway, perform compressions, perform artificial breathing	183	22.9%
	Perform compressions, perform artificial breathing, maintain a patent airway	127	15.9%
	Maintain a patent airway, perform artificial breathing, perform compressions	127	15.9%
	I don’t know	362	45.3%
Which of the following is the number of the medical emergency "Red Crescent"?	993	67	8.4%
	997	531	66.5%
	999	41	5.1%
	I don’t know	160	20.0%
When performing CPR, the compression/ventilation ratio for adults is:	30:2	71	8.9%
	15:2	62	7.8%
	50:5	21	2.6%
	I don’t know	645	80.7%
How long do you think is the duration of a CPR course?	One day	201	25.2%
	One week	80	10.0%
	One month	16	2.0%
	I don’t know	502	62.8%

The assessment of attitudes regarding CPR among the study participants revealed some interesting findings. Among the participants, a significant percentage (65.0%) had not taken a CPR course. However, the majority (84.6%) expressed a strong desire to learn CPR. For those who have not taken a CPR course, reasons varied, including not finding it important (3.9%), leaving it to those specialized in the medical field (3.8%), lack of time (5.1%), and cost concerns (2.0%). Interestingly, the participants showed a positive attitude toward CPR education, with the majority (53.3%) believing that CPR training should be mandatory for all students as a graduation requirement. These findings highlight the importance of promoting and facilitating CPR education to ensure that individuals are equipped with the necessary skills to respond to cardiac emergencies (Table 4).

**Table 4:** Assessment of attitude regarding cardiopulmonary resuscitation among study participants The data have been represented in numbers (N) and percentages (%). CPR: Cardiopulmonary resuscitation.

Parameters	Category	Count (N = 799)	%
If you have taken a CPR course, what encouraged you to take the course?	I have not taken a CPR course	519	65.0%
	It is mandatory for work or graduation requirements	80	10.0%
	Personal benefit	136	17.0%
	Previous experience proved the importance of CPR	29	3.6%
	Other	35	4.4%
Do you want to learn CPR?	No	123	15.4%
	Yes	676	84.6%
If you answer the previous question " NO" please explain why.	I don’t find it important	31	3.9%
	Leave it to those specialized in the medical field	30	3.8%
	I don’t have enough time	41	5.1%
	CPR courses are expensive	16	2.0%
	Other	5	0.6%
	I've answered Yes	676	84.6%
Regarding CPR training, do you think it should be:	Mandatory for all students as a graduation requirement	426	53.3%
	Mandatory before starting a job	94	11.8%
	Mandatory for taking a driver’s license	73	9.1%
	Optional	149	18.6%
	I don’t support the implementation of a CPR training course	57	7.1%

Among the participants, 53.9% responded that they did perform CPR when encountering a situation that required it, while 46.1% did not. For those who did not perform CPR, the reasons varied. Nervousness was reported by 5.9% of participants, while 27.9% mentioned a lack of knowledge about CPR as their reason for abstaining. Additionally, 1.6% expressed a fear of infection via mouth-to-mouth, and 7.8% had concerns about potentially harming the victim. Other reasons were mentioned by 2.9% of participants, and a small percentage (0.3%) stated that they had not encountered a situation that required performing CPR.

Regarding the participants' willingness to perform CPR on different individuals, the majority (74.1%) expressed their readiness to perform CPR whenever needed. However, a small percentage indicated that they would abstain from performing CPR on certain individuals, with 7.3% citing relatives, 6.0% mentioning men, 5.1% referring to women, 4.0% considering children, and 3.5% referring to elderly individuals. When asked about the reasons that would make them abstain from performing CPR, participants provided various responses. Religious reasons, such as gender restrictions, were mentioned by 11.1% of participants, while 18.6% expressed a fear of harming the victim. Lack of confidence in their CPR training was reported by 14.1% of participants, and 6.3% mentioned nervousness as a factor. Other reasons were mentioned by 6.4% of participants. However, the majority (43.4%) affirmed their commitment to perform CPR whenever needed (Table 5).

**Table 5:** Assessment of willingness regarding cardiopulmonary resuscitation among study participants The data have been represented in numbers (N) and percentages (%). CPR: Cardiopulmonary resuscitation.

Parameters	Category	Count (N = 799)	%
If you have encountered a situation that requires performing CPR, did you do CPR?	No	368	46.1%
	Yes	431	53.9%
If you have answered the previous question with "NO" please explain why.	Nervousness	47	5.9%
	No knowledge of CPR	223	27.9%
	Fear of infection via mouth-to-mouth	13	1.6%
	Fear of harming the victim	62	7.8%
	Other	21	2.9%
	I have not encountered a situation that requires performing CPR	2	0.3%
	I answered "Yes"	431	53.9%
When the situation arises, would you abstain from performing CPR on:	Relative	58	7.3%
	Man	48	6.0%
	Woman	41	5.1%
	Child	32	4.0%
	Elderly	28	3.5%
	I would perform CPR whenever needed	592	74.1%
Which of the following reasons would make you abstain from performing CPR?	Religious reasons, e.g., the victim is a woman if you’re a man or vice versa	89	11.1%
	Fear of harming the victim	149	18.6%
	Lack of confidence about your CPR training	113	14.1%
	Nervousness	50	6.3%
	Other reasons	51	6.4%
	I would perform CPR whenever needed	347	43.4%

When asked if they felt their information about CPR was sufficient, a majority of participants (65.2%) responded with "No," indicating a perceived lack of knowledge in this area. Only 6.6% believed they had sufficient information, while 28.2% were unsure.

**Figure 2:**
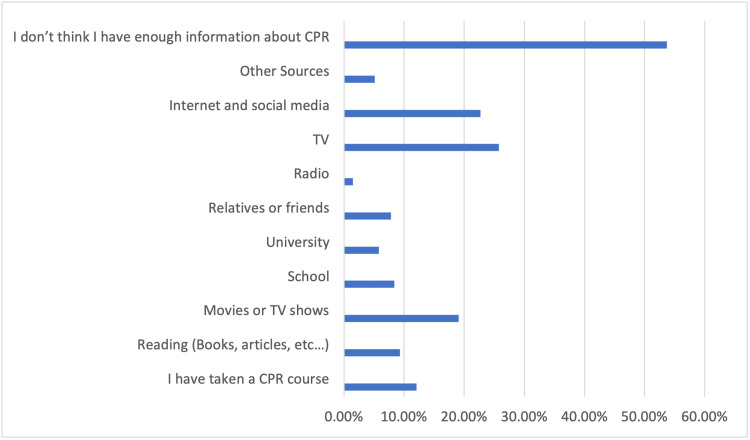
Sources of information regarding cardiopulmonary resuscitation among study participants CPR: Cardiopulmonary resuscitation.

Regarding the sources of information about CPR, participants provided varied responses (Figure 2). Among the options provided, the most commonly selected sources were movies or TV shows (19.1%), followed by TV (25.8%) and the internet/social media (22.7%). A smaller percentage mentioned reading materials (9.3%), school (8.4%), university (5.8%), and relatives or friends (7.8%) as their sources of information. Some participants also mentioned radio (1.5%) and other sources (5.1%). Notably, a significant portion of participants (53.7%) expressed that they did not believe they had enough information about CPR (Table 6).

**Table 6:** Sufficiency and sources of information regarding cardiopulmonary resuscitation among study participants The data have been represented in numbers (N) and percentages (%). CPR: Cardiopulmonary resuscitation.

Parameters	Category	Count (N = 799)	%
Do you think your information about CPR is sufficient?	No	521	65.2%
	Yes	53	6.6%
	I don’t know	225	28.2%
If you have any information about CPR, please indicate the sources of your information (You can choose more than one source):	I have taken a CPR course	97	12.1%
	Reading (books, articles, etc.)	74	9.3%
	Movies or TV shows	153	19.1%
	School	67	8.4%
	University	46	5.8%
	Relatives or friends	62	7.8%
	Radio	12	1.5%
	TV	206	25.8%
	Internet and social media	181	22.7%
	Other sources	41	5.1%
	I don’t think I have enough information about CPR	429	53.7%

**Figure 3:**
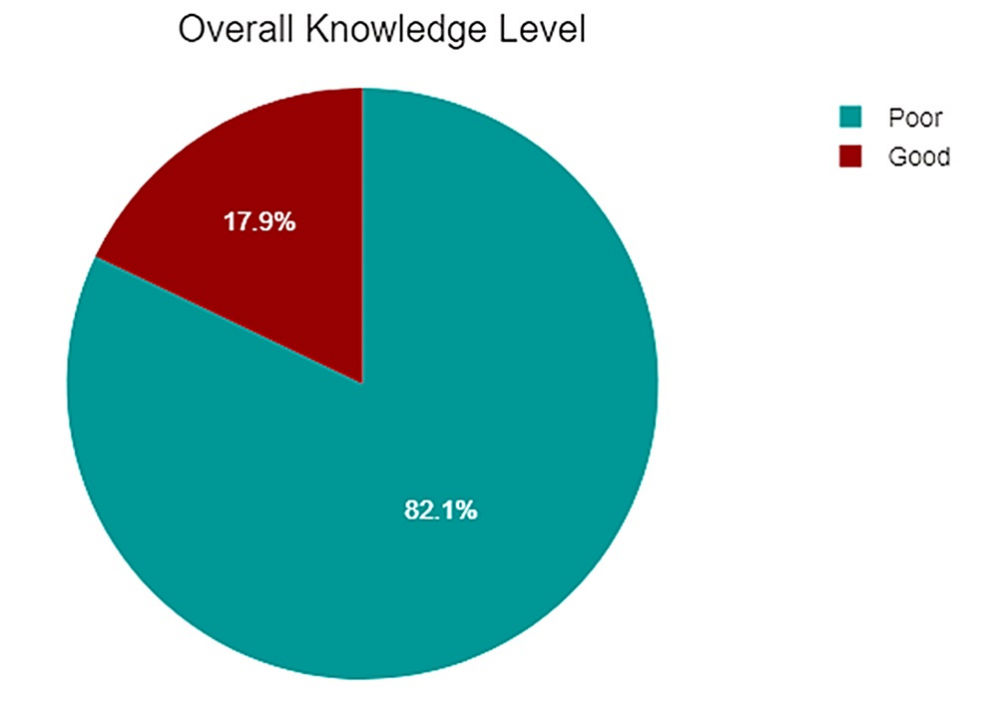
The overall knowledge level regarding cardiopulmonary resuscitation among study participants

The overall knowledge level regarding CPR among the study participants was assessed, with a maximum score of 6. The participants achieved a mean score of 1.44 with a standard deviation of 1.14. Based on the scores, it was found that a majority of participants (82.1%) had a poor level of knowledge about CPR. Conversely, 17.9% of participants were classified as having a good level of knowledge (Figure 3 and Tables 7, 8).

**Table 7:** The overall knowledge level regarding cardiopulmonary resuscitation among study participants The data have been represented in numbers (N) and percentages (%).

Parameter	Category	Count (N = 799)	%
The overall knowledge level	Poor	656	82.1%
	Good	143	17.9%

**Table 8:** Total knowledge score

Parameter	N	Minimum	Maximum	Mean	Std. deviation
Total knowledge score	854	0.00	6.00	1.44	1.14

The analysis revealed that the percentage of participants with a good knowledge level ranged from 12.6% to 35.7% across different age groups. The p-value of 0.038 suggests a weak association between age grouping and the overall knowledge level regarding CPR.

Furthermore, there were no significant associations between these factors and the overall knowledge level regarding CPR. The p-values obtained for gender, region, educational status, employment status, and monthly income were 0.373, 0.102, 0.396, 0.302, and 0.399, respectively. The results indicate that only age grouping plays a significant role in determining the overall knowledge level regarding CPR among the participants (Table 9).

**Table 9:** Factors associated with the overall knowledge level regarding cardiopulmonary resuscitation among study participants

Sociodemographic data		Overall knowledge level				P-value
		Poor		Good		
		Count	%	Count	%	
Gender	Male	267	40.7%	64	44.8%	0.373
	Female	389	59.3%	79	55.2%	
Age grouping	18-25 years	190	29.0%	51	35.7%	0.038
	26-30 years	86	13.1%	18	12.6%	
	31-35 years	64	9.8%	22	15.4%	
	36-40 years	100	15.2%	18	12.6%	
	41-50 years	139	21.2%	20	14.0%	
	51-60 years	57	8.7%	14	9.8%	
	More than 60 years	20	3.0%	0	0.0%	
Region	Central Region	121	18.4%	16	11.2%	0.102
	Western Region	139	21.2%	30	21.0%	
	Northern Region	151	23.0%	36	25.2%	
	Southern Region	166	25.3%	48	33.6%	
	Eastern Province	79	12.0%	13	9.1%	
Educational status	Primary	8	1.2%	0	0.0%	0.396
	Intermediate	9	1.4%	4	2.8%	
	High school	202	30.8%	43	30.1%	
	University	437	66.6%	96	67.1%	
Employment status	I work but not in the medical field	329	50.2%	72	50.3%	0.302
	I am a student but not in the medical field	111	16.9%	31	21.7%	
	I don’t work	216	32.9%	40	28.0%	
Monthly income	Less than 2000 SAR	212	32.3%	54	37.8%	0.399
	2000-10,000 SAR	246	37.5%	51	35.7%	
	10,000-20,000 SAR	168	25.6%	35	24.5%	
	More than 20,000 SAR	30	4.6%	3	2.1%	
Do you have a current or past family member diagnosed with heart disease (myocardial infarction "heart attack," heart failure, congenital heart disease "birth defects," arrhythmia, dyslipidemia "high blood fat")?	No	360	54.9%	68	47.6%	0.111
	Yes	296	45.1%	75	52.4%	

## Discussion

The primary aim of this research was to look into the knowledge, attitude, and willingness of those who had and did not have relatives diagnosed with heart disease to perform CPR and also to establish some of the factors that might influence the knowledge and attitude they have. Furthermore, to try to establish the origin of the knowledge they had. The following discussion will highlight the research implications and the findings that were realized. At the same time, relevant literature will also be employed for comparison purposes.

Many things were revealed by this study. One was that the participants had poor knowledge levels regarding CPR. There were some gaps in how they understood CPR in the population being studied. Essentially, only 40.8% of the participants were able to identify that CPR should be conducted after a person’s heartbeat or heart has stopped. The results derived from our study coincide with the research by Mansour et al., which took place at Qassim University, Saudi Arabia, in which they included 1148 participants, whereby 48.7% were men and 51.3% were female. The study included participants from both medical colleges and non-medical colleges. Their study revealed that the medical students had better knowledge of how CPR is performed compared to the non-medical students. Our study coincides with that of Mansour et al., which noted that CPR knowledge among non-medical students was poor [11]. The medical students were better off in terms of knowledge, attitudes, and practice items. The study conducted at Qassim University, Saudi Arabia, recommended that a BLS course should be added to the university’s program.

Another study by Aldhakhri and Can [12] also looked into CPR knowledge among university students, whereby 31% of the participants did not have prior knowledge of CPR, and those who had previous knowledge of CPR felt that the knowledge they had was not enough, which coincides with our results.

Notably, a study by Alsharari et al. also affirms that knowledge of CPR among university students was not satisfactory despite having a positive attitude toward training [13]. Also, in a study by Alnajjar et al., it was noted that non-medical students had poor knowledge of CPR [14].

In a study conducted by Tamur et al., it was found that the majority of those involved in the study did not have an understanding of CPR training [15]. However, they were willing to learn.

It is noteworthy to mention that the mean knowledge score in the study carried out by Awawdeh et al. was 5.1 (±1.8) out of the possible 10, while the participants in the study recorded a mean of 42.7 (±6.2) in terms of attitude out of the possible 55 points [16], which is a clear indication that people have a positive attitude toward CPR.

Our study has highlighted materials and media individuals use as sources of information on CPR, which are movies or TV shows, TV, and the internet/social media. The study by Aldhakhri and Can affirms this conclusion by noting that television and movies were used as the major sources of information on CPR [12]. The study by Li et al. also affirms that TV shows and movies were the primary sources of CPR information for students [17]. In our study, it was noted that these sources may not give accurate and evidence-based information, and this conclusion coincides with a study conducted by Portanova et al., which noted that TV shows depicted CPR as more effective compared with the actual rates, and this ends up misinforming viewers [18].

The participants in our study noted that they were ready to do CPR on various people, and they would do whatever was necessary. On the other hand, the study by Alhussein et al. noted that legal consequences and fear of causing harm to a given victim would prevent them from performing CPR [6]. In our study, it was indicated that a significant portion of the participants stated that they would not perform CPR on certain individuals, such as a relative, an elderly person, a child, or a woman. This finding is not in line with a study by Alwidyan et al., which stated that most participants were willing to provide standard CPR to a member of their family compared to a hands-on one [19].

Regarding the assessment of attitudes toward CPR among the participants, our study noted that 65.0% of the research participants are yet to take a CPR course. However, most of the participants indicated that they were willing and ready to take the course (84.6%). Those who were not interested in the course had their reasons; some of them included finding the course unnecessary, lack of time, and concerns about cost, and some felt that the course should be left to those in the medical field. About 53.3% of the participants believed that this course should be made compulsory for all university students before they graduate; this finding is in line with the study by Mansour et al., who recommended that a BLS course should be added to the university’s program [11]. The willingness of people to learn is vital as it will bridge the knowledge gap that exists.

In our study, some factors dictating the overall knowledge level regarding CPR are also analyzed. The study indicated that only age plays a significant role in dictating an individual’s knowledge level. Other factors such as gender, region, educational status, and employment status do not dictate the overall knowledge level. On the other hand, the study by Mansour et al. revealed that most female students who were in medical colleges had better knowledge than their male counterparts regarding the location of chest compression. Notably, the p-value was <0.05 [11]. However, it is vital to note that the researchers found no significant difference between participants of both genders with regard to most of the CPR practices, and this is a clear indication that the two studies align on the issue of gender.

Additionally, the presence of relatives suffering from a history of heart disease does not appear to have a significant influence on the knowledge level of individuals. On the other hand, the study by Mansour et al. revealed that most female students who were in medical colleges had better knowledge than their male counterparts regarding the location of chest compression. Notably, the p-value was <0.05 [11]. However, it is vital to note that the researchers found that there was no huge difference between participants of both genders with regard to most of the CPR practices, and this is a clear indication that the two studies align on the issue of gender. In contrast to our study, Teng et al.'s study conducted in South China found a significant association (p < 0.001) between knowledge regarding CPR and the presence of relatives with heart disease. Specifically, individuals who had relatives with heart disease demonstrated higher knowledge of CPR compared to those without such relatives. This finding contrasts with our study, which implies a poor knowledge of CPR among both groups regardless of the presence of relatives with heart disease [4].

The studies have limitations, one of them being that they are reliant on online self-reported data. Another one is that our study did not address the use of automated external defibrillator (AED) in BLS, which would be a starting point for future studies to investigate. The studies acknowledge that there is a need for more training on CPR so that the knowledge gap can be addressed. Through training programs on this essential procedure, a culture where the masses are ready to handle a cardiac emergency will end up being promoted.

## Conclusions

The findings demonstrated gaps in knowledge, with misconceptions regarding CPR. However, participants generally expressed a positive attitude toward CPR education and a willingness to learn. Factors such as age showed a weak association with knowledge level, while gender, region, education, employment, and family history of heart disease did not significantly impact knowledge. The study highlighted the need for improved CPR education and awareness. Hence, we recommend CPR courses to be implemented as a graduation prerequisite. In addition to that, linking some government services to a first aid course would positively impact the general population's practices in CPR and other emergencies.
